# When the Shot Backfires: Demonstrating How Science Communication Can Fail

**DOI:** 10.1177/01461672251370004

**Published:** 2025-10-28

**Authors:** Yashvin Seetahul, Tobias Greitemeyer

**Affiliations:** 1 University of Innsbruck, Institut für Psychologie, Tyrol, Austria

**Keywords:** belief update, motivated reasoning, science communication, violent video games, stigmatization, psychological reactance, bias

## Abstract

We examined how scientific research influences the beliefs of those most invested in the research. We focused specifically on violent video game (VVG) and aggression research and tested the hypothesis that non-players and non-habitual players would update their beliefs based on the study conclusions, whereas more habitual players would not. Participants read abstracts with either a positive effect or a null effect. Beliefs about the effect of VVGs on aggression were measured before and after reading. Results in both studies (overall *N* = 1,576) showed that at low VVG exposure levels, participants in both conditions updated their beliefs to align with the research. However, at high VVG exposure levels, participants maintained their belief that VVGs do not increase aggression, and at very high VVG exposure levels, positive-effect studies led participants to believe the opposite: that VVGs reduce aggression. We interpret this pattern of response as suggesting a process of psychological distancing, whereby individuals defensively shift their beliefs away from threatening claims. These findings highlight the need to understand how scientific information is received and to develop strategies that prevent reinforcing misconceptions.

Psychological research serves two distinct but interconnected purposes, each with its own target audience. On the one hand, it cumulatively advances theoretical understanding by uncovering fundamental principles of human behavior, cognition, and emotion, primarily targeting a scientific readership. On the other hand, it addresses practical, real-world issues by informing public policy, guiding clinical and organizational practices, and shaping the decisions and behaviors of the lay public. Given that psychological concepts often directly touch on individuals’ beliefs and actions, the claims in this scientific field can have an immediate, personal impact on lay readers in ways other sciences might not.

However, this dual role of psychological research presents unique challenges, particularly in how laypeople interpret and react to scientific findings. While researchers are expected to critically assess the methodology, validity, and limitations of the studies they read (Lakens, 2022, chapter 16), lay readers typically lack this specialized expertise. This disparity raises a critical question: How do laypeople update their beliefs when faced with psychological research, especially when the findings contradict their preconceptions or personal interests? Despite researchers’ ethical obligations to present their conclusions objectively, the inherent complexity of psychological findings, coupled with the cognitive biases, heuristics, and firmly held beliefs that lay readers bring to the table, can lead to misinterpretations. Ideally, we would expect individuals to update their beliefs in response to scientific information, as rational decision-makers should. However, motivational and cognitive biases often prevent this, leading people to resist or reject scientists’ claims. As a result, despite encountering evidence that clearly challenges their worldviews, they may maintain their beliefs or even become more convinced of them.

The present two experiments examined the extent to which scientific evidence influences belief updating among individuals with varying personal stakes in the subject matter. Using violent video game (VVG) and aggression research as a case study, we hypothesized that non-players and non-habitual players would adjust their beliefs in accordance with the findings, whereas more frequent players would exhibit resistance to change. This hypothesis is based on previous research showing that there are at least four key threats, namely threats to freedom, threats to consistency, threats to self-concept, and threats to social identity, that can independently or interactively bias how individuals (here, VVG players) respond to disconfirming scientific information.

## Motivational Threats That Shape How People Process Scientific Communication

When individuals perceive that something—such as a persuasive message—threatens or eliminates their freedom to think, behave, or, most relevantly, engage in a valued activity, they experience a motivational state known as psychological reactance (Brehm, 1966; Wicklund & Brehm, 1968). This state is marked by a drive to restore the threatened freedom and is typically expressed through an intertwined combination of anger and counter-arguing cognitions (Dillard & Shen, 2005).

Similarly, when individuals hold two or more cognitions (e.g. beliefs, memories, and attitudes) that conflict, they experience a motivational state—known as cognitive dissonance (Festinger, 1957)—that drives them to restore internal consistency (Festinger & Carlsmith, 1959; Harmon-Jones & Harmon-Jones, 2007). Scientific communication can elicit cognitive dissonance when it presents claims that contradict individuals’ prior beliefs—particularly on topics that are morally, personally, or politically significant. Dissonance is more likely to arise when a message is highly discrepant with one’s attitudes (Bochner & Insko, 1966), and the more a message implies personal involvement, the greater the dissonant arousal it tends to produce (McGrath, 2017; Stone & Fernandez, 2008).

Moreover, when individuals encounter scientific information that implies a shortcoming in their competence, morality, or overall adequacy, it can threaten their self-concept—a global sense of who they are and how they wish to be seen. For example, according to self-affirmation theory (Steele, 1988), people are motivated to preserve a positive sense of their self-concept, and when it is challenged, they engage in psychological strategies to restore it, such as biased information processing.

Finally, scientific communication can threaten individuals’ sense of self by targeting their social identity—the aspect of self-concept derived from group memberships. According to social identity theory (Tajfel & Turner, 1979), people define themselves not only as individuals but also as members of social groups, and they are motivated to view these groups as distinct, morally good, and competent. When a piece of information implies that one’s group is inferior, irrational, or morally suspect, it threatens this collective self-image and elicits motivated resistance aimed at defending the group—and by extension, the self.

## Responses to Threatening Scientific Claims

Individuals who encounter scientific information that threatens their freedom, consistency, self-concept, or social identity often engage in motivated strategies to defend against the discomfort such threats produce. Rather than accepting the message, threatened individuals have been shown to adopt a range of protective responses. Four common strategies include favoring confirming information when confronted with conflicting evidence, selectively seeking belief-consistent information, derogating the source of disconfirming information, and psychologically distancing themselves from threatening claims.

A common response to threatening scientific information involves the biased evaluation of evidence. When individuals are presented with a combination of belief-consistent (i.e., that supports their prior views) and belief-inconsistent claims, they tend to give greater credence to belief-consistent information while discounting or scrutinizing opposing evidence. This tendency—often labeled *confirmation bias* or *biased assimilation*—involves treating belief-consistent evidence as more valid, compelling, or methodologically sound, while simultaneously downplaying or rejecting belief-inconsistent information. A classic demonstration comes from Lord et al. (1979), who showed that participants evaluating mixed evidence about capital punishment judged the study that supported their position as more credible, regardless of its objective quality. A variety of empirical studies illustrate this pattern in real-world science communication. In the domain of climate science, Cook and Lewandowsky (2016) showed that U.S. conservatives rated abstracts supporting anthropogenic climate change as less methodologically sound than those expressing skepticism, even though all abstracts were drawn from the peer-reviewed literature. In the context of vaccination, Hornsey et al. (2018) found that vaccine-hesitant individuals exhibited asymmetric scrutiny: they accepted minor flaws in anti-vaccine research while treating equivalent flaws in pro-vaccine studies as fatal.

Another common strategy for defending against threatening scientific claims is *selective exposure*—the tendency to seek out information that supports one’s existing beliefs while avoiding sources that might challenge them. Multiple psychological mechanisms contribute to this tendency. According to cognitive dissonance theory (Festinger, 1957), people are motivated to reduce the discomfort of holding contradictory beliefs, and exposure to belief-consistent information helps resolve that discomfort. Consistent with reactance theory, when people perceive a message source as controlling or intrusive, they are more likely to avoid it and prefer sources that affirm their sense of freedom (Quick & Kim, 2009). Likewise, when self-concept is at stake, individuals tend to engage with information that protects a valued identity and avoid content that implies personal shortcomings (Sherman & Cohen, 2006).

Another common response to threatening scientific claims is to dismiss the credibility of the messenger. This strategy, known as *source derogation*, involves questioning a communicator’s expertise, trustworthiness, or motives in ways that justify rejecting their claims. Unlike biased evidence evaluation, which selectively weights the merits of the information, or selective exposure, which filters what information is encountered, source derogation targets the communicator, thereby undermining the evidentiary status of the message by association.

A final strategy for defending against threatening scientific claims involves shifting one’s own position further away from the message. This tactic, which we refer to as *psychological distancing*, corresponds to the motivated exaggeration of differences between the self (or one’s group) and a disconfirming source or claim. Rather than rejecting the content outright or attacking the communicator, individuals create distance by emphasizing how dissimilar they are from the people who endorse the view (Prot & Anderson, 2019). They reposition themselves—or their ingroup—on the opposite end of a symbolic divide: “If they believe that, we must believe the opposite.” This strategy reinforces a positive and distinct identity, allowing individuals to maintain integrity without having to assimilate the dissonant information.

While, to our knowledge, the phenomenon has rarely been explicitly presented as a mechanism for belief distortion or biased belief updating, this mechanism is theoretically grounded in self-categorization theory (Turner et al., 1987), which describes how people cognitively organize social information into categories that vary in salience depending on context. When a social category becomes salient, individuals undergo a process of depersonalization, aligning their beliefs and behaviors with those of a prototypical ingroup and distancing themselves from the outgroup (Haslam et al., 2011; Turner, 1985). A core tenet of this theory is the accentuation effect, whereby differences between categories (e.g. ingroup vs. outgroup) are exaggerated and similarities within categories are emphasized, facilitating clearer social meaning and group-based interaction (Haslam et al., 1996; Turner et al., 1994; Van Rooy et al., 2003). Originally demonstrated in perceptual tasks (Tajfel & Wilkes, 1963), this accentuation process operates in social domains as well. The likelihood of such categorization is explained by the meta-contrast principle, which predicts that individuals will classify a set of stimuli (or people) as a group when the perceived intergroup differences outweigh the intragroup differences (McGarty, 1999; Turner et al., 1987; 1994). Through this lens, psychological distancing may be understood as a consequence of motivated group-based cognition: faced with identity-threatening information, individuals are motivated to reaffirm their ingroup identity by cognitively locating the source of the threatening information in an outgroup and then maximizing perceived difference. In doing so, they preserve self- and group-integrity not by rejecting the claim on its merits, but by symbolically withdrawing from its normative implications.

Psychological distancing can differ from other threat responses in both structure and consequence. Whereas biased evidence evaluation modulates how seriously the message is taken, and source derogation questions who is entitled to speak, distancing shifts where the self or group stands in relation to the message. This shift often involves increased attitude extremity, outgroup stereotyping, or sharper perceived ingroup-outgroup divides. As a result, the strategy can fuel polarization, making compromise more difficult and hardening opposition to future evidence. Once distance is created, corrective messages are more likely to be filtered through identity-based heuristics, increasing the likelihood of further resistance.

## The Present Research

Most of the experimental literature on biased belief updating relies on “mixed-evidence” paradigms following Lord et al. (1979)’s approach, where participants are simultaneously presented with one research summary that confirms and another that challenges their prior view. While this type of design is well-suited for revealing motivated biases such as biased assimilation and source derogation, it is less suited for addressing an equally important question: What happens when people encounter a single, internally consistent body of evidence that challenges their beliefs? As Anglin (2019) observes, lay readers rarely encounter rival studies side by side. Instead, they are more likely to encounter “a single pattern of findings” framed as a coherent scientific message (pp. 2–3). Rosman and Grösser (2024) make a complementary point: juxtaposing pro- and counter-attitudinal evidence invites motivated reasoners to cherry-pick the congenial half, thus facilitating biased assimilation and potentially muting other defensive strategies—such as selective exposure or psychological distancing—that are difficult to isolate in such paradigms.

Both Anglin (2019) and Rosman and Grösser (2024) attempted to address this limitation by exposing participants to a unidirectional set of findings. Anglin asked U.S. MTurkers to read fictitious research summaries that either uniformly supported or opposed their stance on polarizing issues such as religion, gun control, or the death penalty’s deterrent effect. Rosman and Grösser presented German adults with fictitious summaries that either endorsed or questioned the clinical efficacy of acupuncture. In both cases, participants’ beliefs shifted in the direction of the evidence, demonstrating *alignment* with the claim. On the surface, these findings suggest that clear, consistent messages can overcome the usual barriers to persuasion.

We view this conclusion as premature for two reasons. First, it may be that in their studies, the topics may have involved limited personal stakes. Rosman and Grösser explicitly excluded participants who had received acupuncture in the past decade—likely omitting those most personally invested in the topic. Likewise, Anglin notes that the topics in their study may not have been “particularly threatening” to participants’ identities (pp. 195–196). This matters, because defensive biases are most likely to emerge when individuals feel personally or ideologically threatened. And second, the results of their studies could have been biased by repetitive belief measures that were too transparent. Both studies relied on explicit self-report items administered before and immediately after the reading task. This repetition, combined with the direct and obvious phrasing of the items, likely heightened transparency and may have induced demand compliance. Participants may have inferred the researchers’ expectations and adjusted their responses accordingly (Coles et al., 2023; Nichols & Maner, 2008).

Hence, to our knowledge, no prior study has combined (a) a single, internally-consistent set of claims, (b) a continuously-measured personal-stake moderator, and (c) a low-transparency belief metric. To build on this foundation while addressing these limitations, the present research adopts three design features. First, similarly to Anglin (2019) and Rosman and Grösser (2024), we use unidirectional sets of findings. In our studies, participants read a homogeneous set of research abstracts. Second, we measured participants’ position on a continuous dimension related to the personal relevance of the topic and the extent to which the claims might be threatening. Rather than dichotomizing topic relevance, we measured it continuously, enabling us to test for graded defensive bias. Third, we employed a subtle, non-repetitive belief measure, with a novel task designed to minimize transparency and demand characteristics.

We focused on the long-standing debate regarding VVGs and aggression—a domain particularly well-suited for this investigation. With more than 400 empirical studies since the 1970s (Calvert et al., 2017) and over 3.3 billion players globally (Howarth, 2024), video games are more than a leisure activity—they are embedded in daily routines, social lives, and identities (Anderson, 2020; De Grove et al., 2015; Hazel et al., 2022; Hilgard et al., 2013), highlighting the fact that there is widespread personal relevance for this topic. Furthermore, there is persistent scientific controversy. Despite decades of research, meta-analyses and primary studies continue to reach conflicting conclusions. The field is divided between scholars claiming VVGs increase aggression and those detecting no effect (Elson et al., 2015; Greitemeyer & Mügge, 2014; Hilgard, Engelhardt, Bartholow, et al., 2017; Hilgard, Engelhardt, & Rouder, 2017; Mehta & Lakens, 2023). This enduring uncertainty keeps the issue salient and contested.

Importantly, the VVG debate plausibly implicates all four motivational threats described earlier. First, it evokes threats to freedom: policy proposals such as bans, warning labels, or age restrictions imply that gameplay is socially harmful, which threatens a valued leisure freedom. Consistent with this, players show reactance and strategic self-control when they know their aggression is being measured (Bender et al., 2013; Seetahul & Greitemeyer, 2024). Second, it involves threats to consistency: many long-term gamers view their own experience as harmless, so research suggesting increases in aggression creates a conflict between belief and evidence. Gamers, in fact, report believing that VVGs are harmless (Greitemeyer, 2014). Third, the issue threatens their self-concept: for many players, gaming is a source of pride, competence, or creativity. Prior work shows that gaming supports self-image as capable or strategic (C. G. Anderson, 2020; De Grove et al., 2015). Claims that games foster aggression imply a moral or character flaw in those who play them: if VVGs increase aggression, then VVG players are aggressive—which is an undesirable characteristic in society. Fourth, it elicits threats to social identity: “gamer” is a salient ingroup label, and negative research findings are often perceived as stigmatizing the group. As a consequence, gamers tend to discredit researchers who publish harmful-effect findings and report feeling collectively targeted (Nauroth et al., 2014, 2015).

Consistent with this threatening landscape, prior work has documented several defensive responses among VVG players. Exposure to both pro- and anti-VVG findings increases conviction in one’s prior view (Greitemeyer, 2014), demonstrating biased assimilation. Players avoid articles suggesting VVGs increase aggression (Seetahul & Greitemeyer, 2025b), demonstrating selective exposure. And researchers who claim that VVGs increase aggression are rated as less competent, less friendly, and more biased (Nauroth et al., 2014; Seetahul & Greitemeyer, 2025b), demonstrating source derogation. Taken together, these dynamics make the VVG debate an ideal test case for our central question: Can a single, coherent set of disconfirming findings sway beliefs when the evidence can be perceived as threatening?

To address this, we conducted two pre-registered experiments in which participants read abstracts describing either a positive effect or a null effect of VVGs on aggression. Beliefs about the relation between VVGs and aggression were subtly measured before and after reading the abstracts, and VVG exposure was assessed along a continuous dimension. We expected that the influence of the type of abstracts on belief update would be moderated by VVG exposure, that is, we predicted that abstracts would be more successful at convincing readers at low levels of VVG exposure compared to high levels. The design, hypothesis, and analysis plan for both studies were pre-registered, and all registrations (including documented deviations), data, materials, supplemental materials, and fully reproducible analysis code are openly available on the OSF repository (https://osf.io/p7cds/).^1^

## Study 1

### Method

Study 1 was conducted online from August 8th, 2023 to August 24th, 2023. The participants for this study were recruited using Prolific (www.prolific.com) with English as a fluent language and 95% approval ratings as filters. The study was deceptively presented to participants as an examination of individual preferences regarding scientific communication. They were told that our purpose was to obtain their opinions regarding two distinct types of scientific communication. To maintain the study’s deceptive element, participants were led to believe they would be randomly assigned to one of several topics regarding societal issues, including “Global Warming and Climate Change Denial,” “The Importance of Vaccines and Conspiracy Theories,” “The Effects of Video Games,” “Workplace Stress and Burnout,” and “Addictions and Substance Abuse.” After completing demographic and personality questionnaires, participants were then informed that their assigned topic would specifically be “The Effects of Video Games” as a result of the alleged random selection.

The sample size was determined a priori by a power analysis conducted using *R* (R Core Team, 2023), on *R Studio* (Posit Team, 2023), with the *InteractionPower* package (Baranger et al., 2023) for a continuous predictor (i.e. VVG exposure), a two-level moderator (i.e. “Positive effect” vs. “Null effect”), and a continuous outcome (i.e. belief update). The power analysis was conducted assuming small effect sizes (*r* = .10) for both main effects, the interaction effect, and the correlation between the two predictor variables. A sample size of *N* = 750 was sufficient to provide 80% power (1−β err probability) to detect the interaction given these parameters (with an α err probability of .05). To account for potential exclusion of data due to any data quality issue, we increased the sample size by 5%, resulting in *N* = 787.5 (rounded to 788), thus providing us with a statistical power of 81.2% to detect the moderation effect.

The *N* = 788 participants were from 34 different countries. The mean age of the sample was *M* = 29.45 (*SD* = 9.1), *n* = 421 were females, *n* = 363 were males, *n* = 1 reported being intersex, *n* = 1 reported being non-binary, and *n* = 1 reported being queer.

After being informed that they would read scientific communications about the effect of video games, participants were asked to report the average number of days per week they have spent playing games of nine video game genres, over the past 5 years. The answers ranged from 0 days to 7 days. The nine genres were: Action games, Adventure games, Action-Adventure games, Role-Playing games, Strategy games, Simulation games, Sandbox games, Massively Multiplayer Online Role-Playing games, and Sport games.

**Figure 1.**
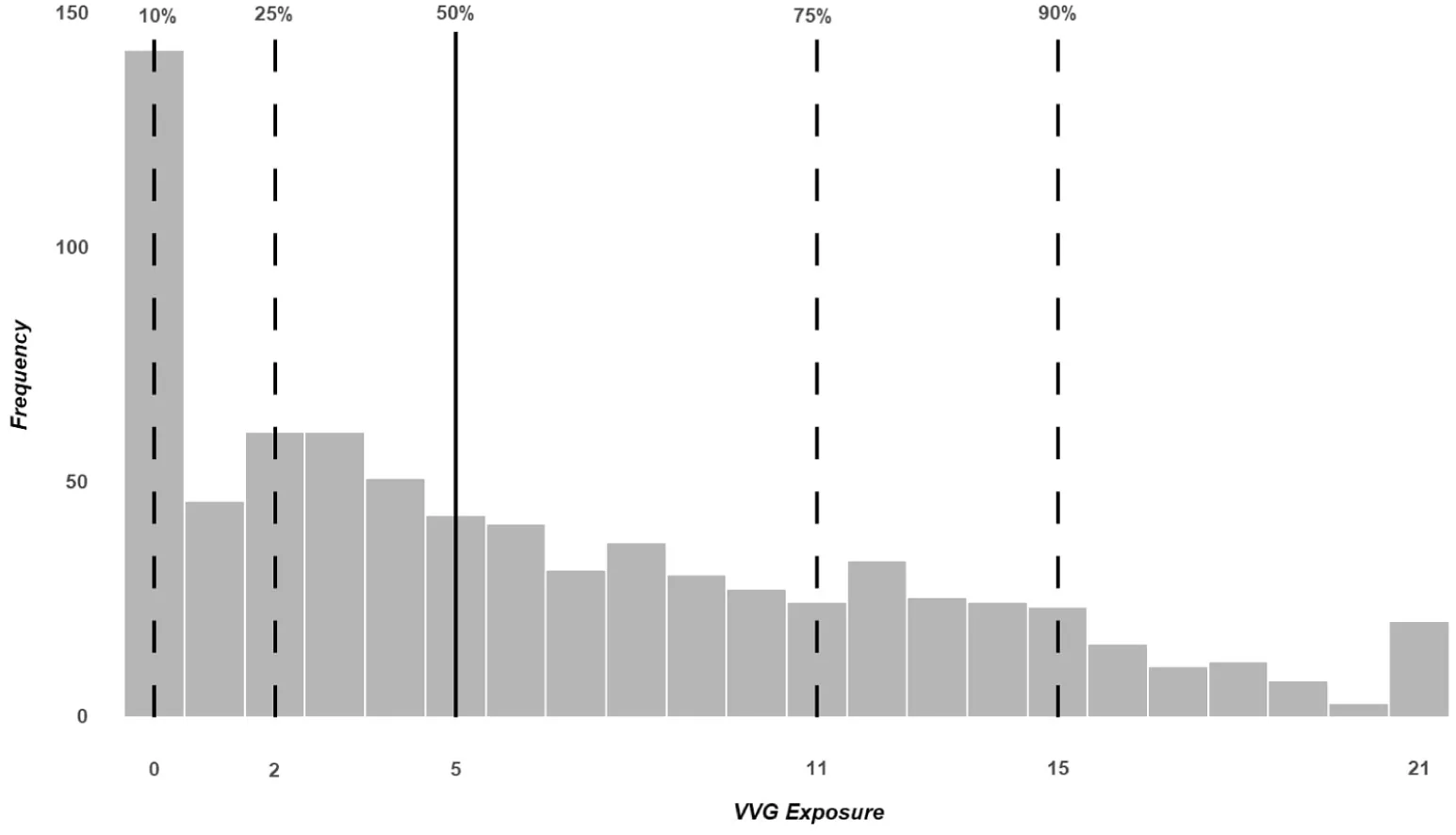
Histogram of the distribution of VVG exposure scores (Study 1). *Note*. The solid vertical line represents the median value and the four dashed lines represent the 10th, 25th, 75th, and 90th percentiles.

VVG Exposure (*M* = 6.56, *SD* = 5.84; see Figure 1 for the full distribution) was computed by calculating the sum of the frequency of game play for Action, Adventure, and Action-Adventure video games. This measure has worked successfully in the past (Seetahul & Greitemeyer, 2024, 2025a), and similar genre-based measures have also been used in the literature (e.g. Richmond & Wilson, 2008). The focus on Action, Adventure, and Action-Adventure video games is due to the prevalence of violence as a central gameplay element in these genres. Progress in these games typically requires engaging in physical aggression against characters or other players, an aspect that is deeply integrated into the games’ thematic and narrative context. This is in contrast to other genres where violent actions may not be as central, but rather a potential means to an end.^2^

Participants were informed that they would read two scientific communications. We presented the abstract of a meta-analysis, followed by the abstract of an empirical primary study. Since we presented the study as being about the evaluation of different types of scientific communication, we included filler questions after each scientific communication to ask participants about their opinions and to evaluate the abstract they just read.

Participants were randomly assigned to the “Positive effect” condition (*n* = 426), in which both the meta-analysis and the primary study concluded that VVGs increase aggression, or to the “Null effect” condition (*n* = 362) in which both the meta-analysis and the primary study concluded that no effect of VVGs was found on aggression.

In the “Positive effect” conditions, for each participant, the meta-analysis was randomly selected between Anderson et al. (2010), Burkhardt and Lenhard (2022), and Calvert et al. (2017). The primary study was randomly selected between Bartholow and Anderson (2002), Carnagey and Anderson (2005), and Konijn et al. (2007).

In the “Null effect” conditions, for each participant, the meta-analysis was randomly selected between Drummond et al. (2021), Ferguson (2015), and Hilgard et al. (2017). The primary study was randomly selected between Engelhardt et al. (2015), Ferguson et al. (2008), and Ferguson and Rueda (2010).

Participants were informed that “aggressivity” or “aggressiveness” refers to a mental and physical state where an individual is more likely to respond aggressively to frustration or provocation (for example, being disrespected, challenged, or insulted) and that the state of being aggressive is not a permanent condition and can be influenced by factors such as stress, mood, and social context. They were further informed that aggressivity could therefore be quantified, for each individual, within each context, as the likelihood of responding aggressively to frustration or provocation.

Participants were then presented with six hypothetical scenarios involving a “typical video game player” playing a video game. These scenarios were crafted to assess participants’ perceptions of how VVG exposure affects aggression, with the following randomly varying parameters:

- *Prior aggressivity*: Each scenario specified a level of aggressivity for the video game player before playing the video game, with three “low” levels (15%, 25%, 35%) and three “high” levels (65%, 75%, 85%). The order of the six levels was randomized across the six scenarios.
- *Violent content within the game*: The amount of violence in the game varied. Each scenario indicated a value that was randomly selected between 20%, 50%, and 80%, representing “low violence,” “moderate violence,” and “high violence,” respectively.
- *Duration of play*: The time spent playing also varied. Each scenario indicated a value, which was randomly selected between 1 hour, 3 hours and 30 minutes, or 6 hours, representing a “low amount of time spent playing,” “moderate amount of time spent playing,” and “high amount of time spent playing,” respectively.
- For each scenario, participants were asked to estimate the aggressivity level of the “typical player” after playing (i.e. the likelihood of responding aggressively to frustration or provocation), on a slider scale from 0% to 100%, *given* the specified prior aggressivity level, the amount of violent content within the game, and the time spent playing.

We used the following three steps to quantify the belief:

- *Player Aggression Change*: For each participant, we calculated the change in aggressivity (Δ_Aggressivity_) for each of the six scenarios, as indicated in Equation (1):

(1) $$\Delta_{Aggressivity}=\;Player\;aggressivity\;-\;Prior\;aggressivity$$

- *Violence Exposure*: For each participant, violence exposure was quantified as the product of the game’s violence level and the duration of play for each of the six scenarios, as indicated in Equation (2):

(2) $$\begin{array}{l} Violence\;Exposure\;=\;Violent\;Content\;\\ \;\;\;\;\;\;\;\;\;\;\;\;\;\;\;\;\;\;\;\;\;\;\;\;\;\;\;\;\;\;\;\;\;\;\;\;\;\;\;\;\;\;\;\;\;\times \;Duration\;of\;Play \end{array}$$

- *Regression Model*: A regression model with ordinary least squares was fitted *for each participant individually* using the values from the six scenarios (i.e. six data points), with Δ_Aggressivty_ as the outcome variable and Violence Exposure as the predictor variable, as indicated in Equation (3):

(3) $$\Delta_{\mathrm{Aggressivity}}=\;\beta 0\;+\;\beta 1(Violence\;Exposure)+\;\varepsilon$$

The slope coefficient β1 from the regression model represents the participant’s belief about the relationship between VVG exposure and aggression.

Participants’ beliefs were measured, as described above, both before and after the experimental manipulation. The belief update was calculated as the difference in β1 values post-manipulation minus pre-manipulation, as indicated in Equation (4):

(4) $$Belief\;update\;=\beta 1_{Post-manipulation}-\;\beta 1_{Pre-manipulation}$$

The average level of belief update was *M* = 0.00 (*SD* = 0.14). A value of 0 represents a lack of belief update, indicating that the articles did not change the participants’ mind. Strong negative values for belief update indicate a strong decrease in the believed relationship, and strong positive values indicate a strong increase in the believed relationship.

This measure, therefore, corresponds to the relationship between VVG exposure and aggression *according to* each participant. This conceptualization focuses on the belief that VVG exposure can *change* aggressiveness rather than completely *generate* it. More precisely, our measure considers that participants may assume that VVG exposure can alter a player’s aggression level: a positive β1 value suggests a belief that VVGs increase aggression, while a negative β1 implies a belief that VVGs decrease aggression. The absolute value of β1 indicates the strength of this belief.

The use of ordinal least squares to estimate each participant’s level of belief, with individual participant-specific models, can be considered as a method to capture their true underlying belief by disentangling, each time, the estimate from the residual error (e.g. context-specific variability) and the β0 intercept, which would correspond players’ baseline aggressiveness, altering participants’ judgment of the video game player’s post-play aggressiveness. This belief measure captures participants’ perceived causal relationship between VVG exposure and aggression by estimating individualized slopes from responses to parametrically varied vignettes. Rather than asking for explicit agreement with generalized claims, our approach infers beliefs from numeric judgments about hypothetical scenarios, isolating the perceived effect of violent gameplay on post-play aggressiveness. This method avoids the heuristic-driven, socially filtered cognitive processes that contaminate traditional declarative belief measures—such as motivated reasoning, impression management, or consistency pressures.^3^

### Results

All analyses were conducted using *R* (R Core Team, 2023) in *R Studio* (Posit Team, 2023), along with the *emmeans* package (Lenth, 2022), the *effectsize* package (Ben-Shachar et al., 2020), the *correlation* package (Makowski et al., 2020, 2022), and the *TOSTER* package (Caldwell, 2022; Lakens, 2017). The formatting was conducted with the help of the *dplyr* package (Wickham et al., 2022). The data visualizations were obtained with the *ggplot2* package (Wickham, 2016), along with the *patchwork* package (Pedersen, 2025) and the *gridExtra* package (Auguie, 2017).

## Pre-Manipulation and Post-Manipulation Beliefs

**Figure 2.**
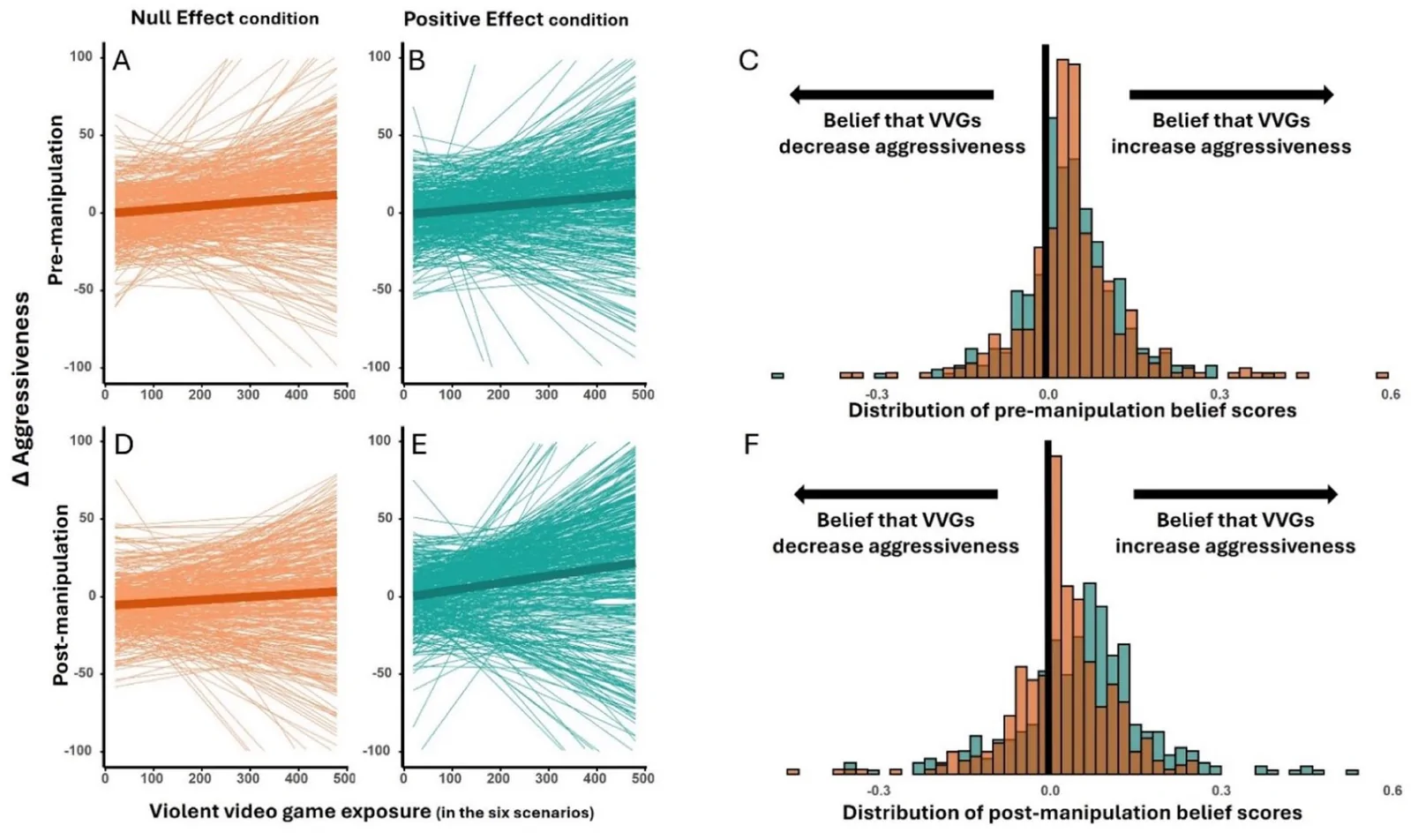
The relationship between VVG exposure and aggressivity according to each participant (in Study 1). *Note*. The individual thin lines in panels A, B, D, and E represent the relationship between VVG exposure and aggression according to each individual participant, and the thick lines represent the average of all participants within the corresponding category. Panel A shows the believed relationship pre-manipulation in the “Null Effect” condition (*M* = 0.04, *SD* = 0.10). Panel B shows the believed relationship pre-manipulation in the “Positive Effect” condition (*M* = 0.03, *SD* = 0.12). Panel C corresponds to the overlapping distribution histograms of the pre-manipulation belief scores (i.e. the slope coefficients from panel A and panel B). Panel D shows the believed relationship post-manipulation in the “Null effect” condition (*M* = 0.01, *SD* = 0.09). Panel E shows the believed relationship post-manipulation in the “Positive effect” condition (*M* = 0.05, *SD* = 0.12). Panel F corresponds to the overlapping distribution histograms of the post-manipulation belief scores (i.e. the slope coefficients from panel D and panel E). Positive scores correspond to the belief that VVGs increase aggressivity, and negative slopes correspond to the belief that VVGs decrease aggressivity.

The average level of pre-manipulation belief was *M* = 0.03 (*SD* = 0.11), with practically identical levels in the “Positive effect” condition (*M* = 0.03, *SD* = 0.12) and the “Null effect” condition (*M* = 0.04, *SD* = 0.10), *t*(784.87) = 0.88, *p* = .379, Cohen’s *d* = .06, 95% CI [−0.08, 0.20] (see Figure 2). A Two-One-Sided-Test (Lakens, 2017) further confirmed the equivalence of the level of pre-manipulation belief in the two conditions for equivalence bounds from Cohen’s *d* = −.05 to Cohen’s *d* = .05, *t*(784.87) = −6.17, *p* < .001. Moreover, the pre-manipulation belief was not related to VVG exposure, *t*(786) = −1.85, *p* = .064, Std. β = −.07, 95% CI [−0.14, 0.004].

The average level of post-manipulation belief was also *M* = 0.03 (*SD* = 0.11), but with significantly different levels in the “Positive effect” condition (*M* = 0.05, *SD* = 0.12) and the “Null effect” condition (*M* = 0.01, *SD* = 0.09), *t*(775.44) = −5.25, *p* < .001, Cohen’s *d* = −0.38, 95% CI [−0.52, −0.24] (see Figure 2). The post-manipulation belief was also significantly related to VVG exposure, *t*(786) = −7.15, *p* < .001, Std. β = −.25, 95% CI [−0.31, −0.18]. Moreover, the post-manipulation belief was predicted by the interaction of VVG exposure and the two conditions, *t*(784) = −6.53, *p* < .001, Std. β = −.44, 95% CI [−0.57, −0.31] (see Figure 2).

In the Null-effect condition, the simple slope never differed from zero across the entire VVG‑exposure range (all |*t*| < 3.0, Bonferroni-adj. ps > .12). In the Positive-effect condition, the simple slope was above zero for low-to-moderate VVG exposure (0–10), statistically indistinguishable from zero for mid-range VVG exposure (11–16), and below zero for heavy VVG exposure (≥ 17).^4,5^

The pre-manipulation belief and the post-manipulation belief were correlated at Pearson’s *r* = .11, 95% CI [0.04, 0.18], *t*(786) = 3.14, *p* = .002.

### Pre-Registered Analysis: The Moderation Effect of VVG Exposure and Article Conclusion on Belief Update

**Figure 3.**
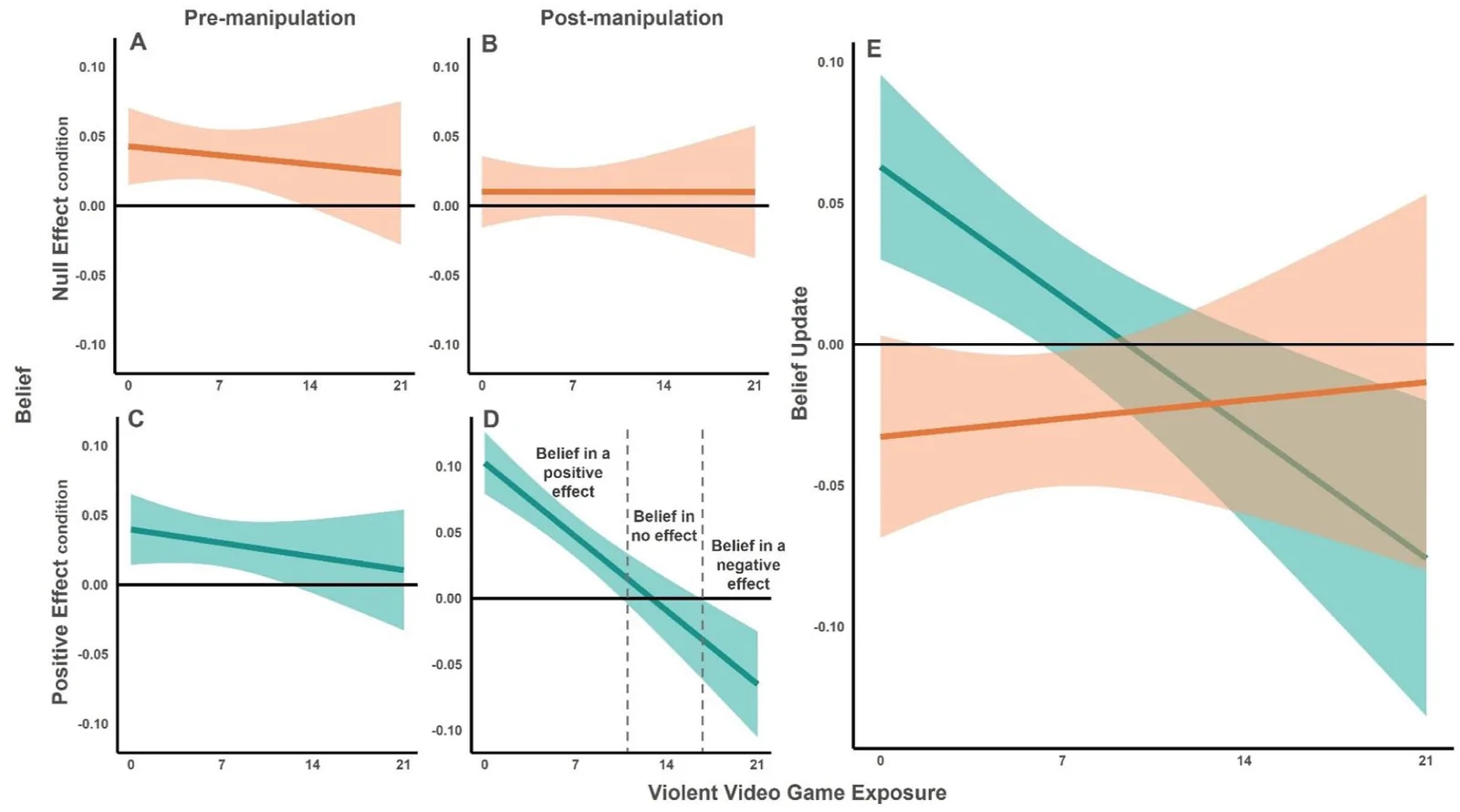
The relationship between VVG exposure, belief, and belief update in the two conditions (Study 1). *Note*. The *x*-axis in the five panels corresponds to participants’ VVG exposure. The *y*-axis on panels A, B, C, and D corresponds to the belief about the relationship between VVG exposure and aggressivity. The *y*-axis in panel E corresponds to the belief update (i.e. the post-manipulation beliefs minus the pre-manipulation beliefs). The thick horizontal line in the five panels corresponds to the zero threshold. Panel A shows participants’ pre-manipulation belief, as a function of VVG exposure, in the “Null effect” condition (Std. β = −.05). Panel B shows participants’ post-manipulation belief, as a function of VVG exposure, in the “Null effect” condition (Std. β = −.0002). Panel C shows participants’ pre-manipulation belief, as a function of VVG exposure, in the “Positive effect” condition (Std. β = −.08). Panel D shows participants’ post-manipulation belief, as a function of VVG exposure, in the “Positive effect” condition (Std. β = −.44). Panel E shows the belief update, as a function of VVG exposure, in “Null effect” condition (Std. β = .04) and the “Positive effect” condition (Std. β = −.27). The bands in all five panels correspond to the Bonferroni-adjusted-95% CIs.

We employed a linear regression model with an interaction term between the condition variable and VVG exposure, and with belief update as the outcome variable.^6^ In support of our hypothesis, the interaction between the condition variable and VVG exposure was significantly related to belief update, *t*(784) = −4.41, *p* < .001, Std. β = −.31, 95% CI [−0.45, −0.17] (see Figure 3). The simple slope for the relationship between VVG exposure and belief update in the “Positive effect” condition was *t*(784) = −5.97, Bonferroni-adjusted- *p* < .001, Std. β = −.27, 95% CI [−0.37, −0.17]. The simple slope for the relationship between VVG exposure and belief update in the “Null effect” condition was *t*(784) = 0.71, Bonferroni-adjusted- *p* = .959, Std. β = .03, 95% CI [−0.08, 0.16].

In the Null-effect condition, updates stayed near zero across the exposure continuum; only very light gamers (2–8) showed a small but significant downward drift (all other points |*t*| < 3.0, adj. ps > .10). In the Positive-effect condition, updates were positive for light gamers (0–6), statistically indistinguishable from zero for mid-range exposure (7–15), and negative for heavy gamers (≥ 16).^7^

### Exploratory Analyses

To examine which type of conceptualization best describes the shifts in belief in the “Positive Effect” condition, we recoded each participant’s pre- and post-β slopes into three alternative categorical outcomes that represent three approaches to conceptualize the changes in participant beliefs (see Table 1): either accounting for *the specific qualitative paths* (i.e. such as from a “positive” belief to a “null” belief), or accounting only for *the net direction of the belief update* (i.e. whether the belief increased, remained unchanged, or decreased), or accounting for *the congruency of the shift* (i.e. whether the belief change consists in an *alignment* with the message that VVGs increase aggression or a *distancing* from that message).^8^ We then fitted three models, one with each type of conceptualization of belief update as the outcome, and each with VVG exposure as the predictor. We then compared the three models to examine which model best described our data.

**Table 1.** Three Categorical Codings Used to Characterize Belief-Shift Patterns.

Type of belief update	Levels	Conceptualization
Shift-trajectory	*9 levels:* Negative ➔ Negative Negative ➔ Null Negative ➔ Positive Null ➔ Negative Null ➔ Null Null ➔ Positive Positive ➔ Negative Positive ➔ Null Positive ➔ Positive	“What matters is the exact *path* from pre to post.”
Shift-direction	*3 levels*: Increase in belief Belief perseverance Decrease in belief	“What matters is only the *net direction* of movement.”
Shift-distancing	*2 levels*: Alignment Distancing	“What matters is whether the update *aligns* with or *moves away* from the message.”

*Note*. Belief slopes (β) were first classified as Negative (β < –.05), Null (–.05 ≤ β ≤ .05), or Positive (β > .05).^9^ These region labels were then combined to create the nine Shift-Trajectory categories. Shift-Direction collapses those nine paths into the overall vector of change with three possible directions. Shift-Distancing treats an update as aligned if it moves in the same direction as the experimental message (or remains consistent when already congruent) and as distanced if it moves in the opposite direction.

We implemented the three models with the belief update patterns as the outcome and VVG exposure as the predictor using the VGAM package (Vector Generalized Linear and Additive Models; Yee, 2015) in order to have the appropriate link family for each model within the same statistical framework: a nominal multinomial model for Shift-Trajectory (m1), a cumulative logit model for Shift-Direction (m2), and a binary logistic model for Shift-Distancing (m3), thereby facilitating the model comparisons.

**Figure 4.**
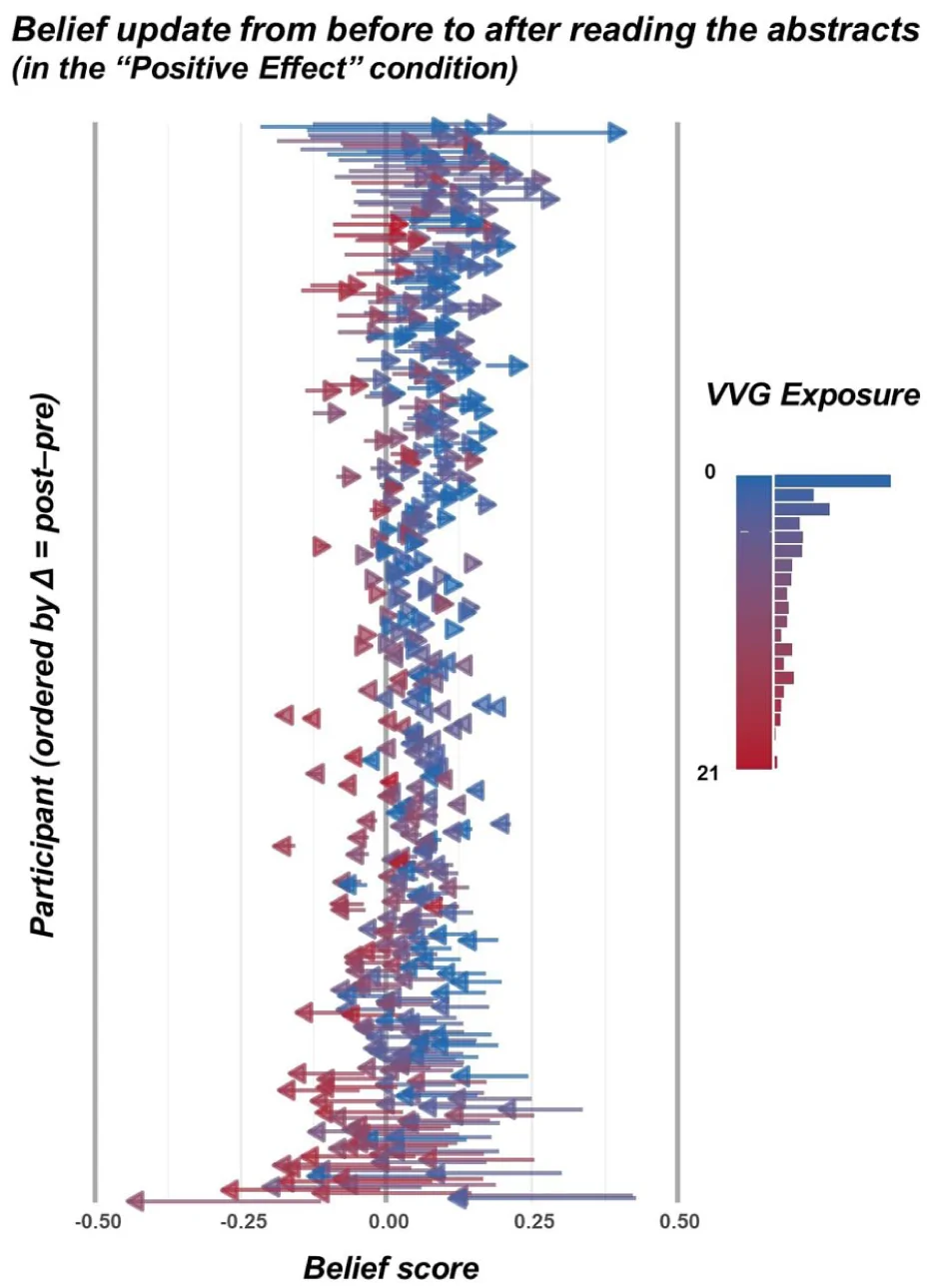
Individual belief updates in the “positive effect” condition. *Note.* Each arrow represents one participant (*n* = 426) in the “Positive Effect” condition (i.e. a participant who read article abstracts claiming that VVGs increase aggression). The tail of each arrow marks the pre-reading belief score; the head marks the post-reading score. Arrows pointing right indicate beliefs shifting toward the notion that VVGs increase aggression (“*alignment*”). Arrows pointing left indicate beliefs shifting away from the notion (“*distancing*”). Participants are vertically ranked by the belief change, with the strongest alignment at the top and the strongest distancing at the bottom (the two most extreme positive and two most extreme negative shifts are removed from this data visualization for clarity). Arrow colors correspond to continuous VVG exposure scores (blue = VVG score of 0; red = VVG score of 21). The vertical histogram in the legend corresponds to the distribution of VVG exposure scores.

**Figure 5.**
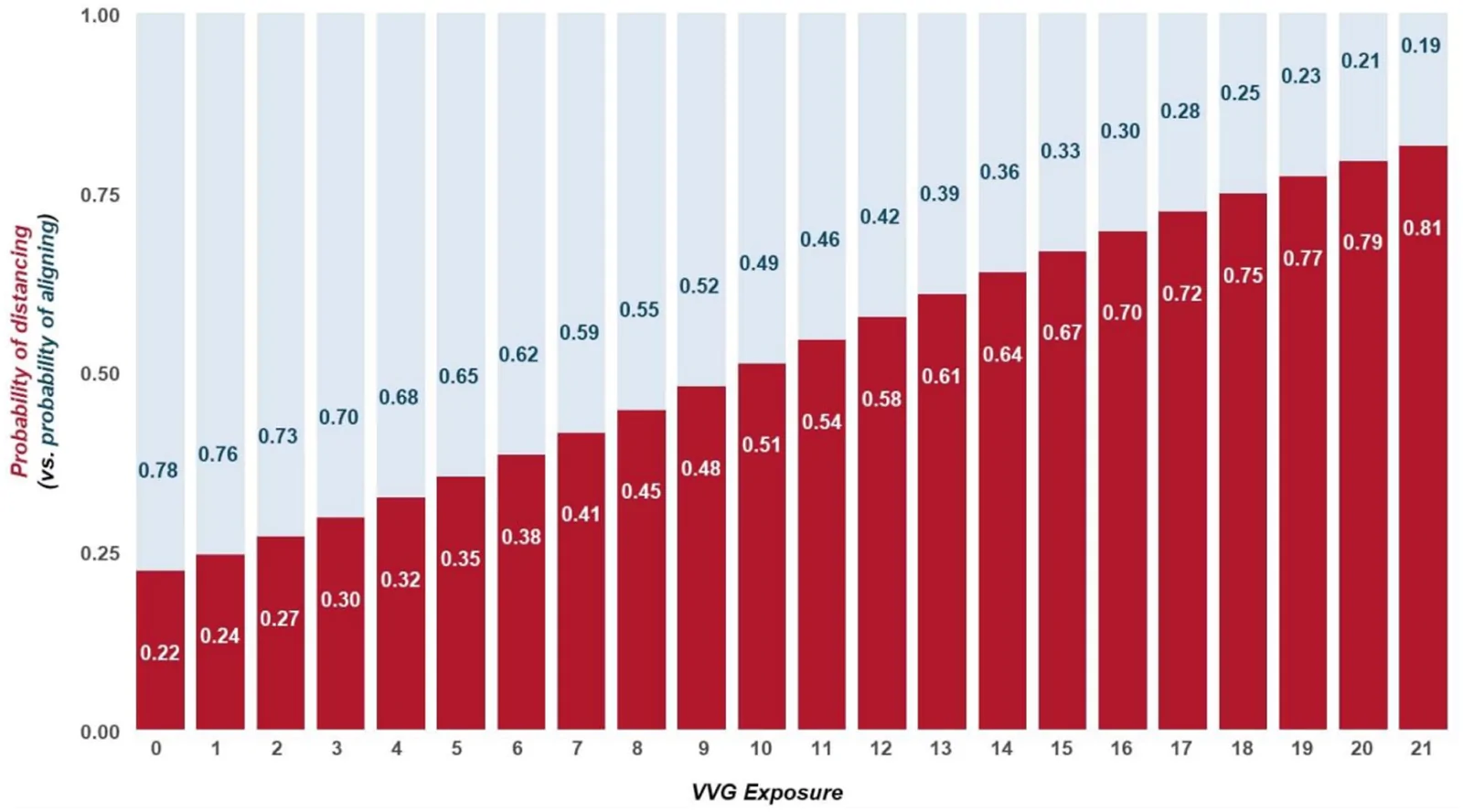
The probability of distancing from the claim (vs. the probability of aligning) as a function of VVG exposure. *Note.* The figure displays the predicted probability of participants distancing from the claim (vs. aligning with it) as a function of VVG exposure, among participants assigned to the “Positive effect” condition (*n* = 426). Predicted probabilities are shown for each integer level of VVG exposure (0–21). Probabilities were derived from a binary logistic-regression model in which VVG exposure was entered as a continuous predictor of distancing from the claim (reference category = aligning). For every exposure level, the fitted logit was first calculated and then converted to a probability with the inverse-logit transformation. The model yielded an odds ratio of 1.14 (*p* < .001), indicating a 14% increase in the odds of distancing for each additional unit of VVG exposure. Numeric labels inside the bars are the rounded predicted probabilities.

The results of m1 indicated that, relative to the “Null ➔ Null” reference level, VVG exposure was associated with four specific transitions: Negative ➔ Null (*OR* = 1.11, *p* = .025), Null ➔ Negative (*OR* = 1.25, *p* < .001), Null ➔ Positive (*OR* = 0.90, *p* < .001), and Positive ➔ Negative (*OR* = 1.37, *p* = .017). Other paths were non-significantly different from the reference level. The results of m2 indicated that, relative to belief perseverance (i.e. no change), VVG exposure reduced the odds of an increase (*OR* = 0.94, *p* = .008), and raised the odds of a decrease (*OR* = 1.11, *p* < .001). The results of m3 indicated that each unit increase in VVG exposure increased the odds of moving away from the message (i.e. the claim that VVGs increase aggression) by 14%, *OR* = 1.14, *p* < .001 (see Figure 4). Hence, at a VVG exposure level of 0, the probability of aligning to claim is 78% versus a 22% probability of distancing, whereas at a VVG exposure level of 21, the probability of aligning is 19% versus an 81% probability of distancing (see Figure 5).

As summarized in Table 2, the binomial Shift-Distancing model achieved the lowest AICc (1,123) and BIC (1,140), the smallest RMSE (2.22), and the lowest residual σ (1.11). Both multinomial specifications showed substantially higher information-criterion values (Δ AICc ≥ 56) and larger errors. These indices indicate that the simplest binary coding of Shift-Distancing captures the belief shifts most efficiently, and that finer distinctions regarding the exact post-belief location add complexity without improving explanatory power. 

**Table 2.** Model Comparison Metrics.

Model	Pattern	AICc	Δ AICc	BIC	RMSE	Residual σ
m1	*Shift-trajectory*	1,179	56	1,215	2.87	1.39
m2	*Shift-direction*	1,245	122	1,281	6.02	1.69
m3	*Shift-distancing*	1,123	0	1,140	2.22	1.11

### Discussion

The results of Study 1 support our hypothesis. Prior to reading, on average, participants believed in a very small relationship between VVGs and aggression (that did not significantly differ from zero). At low levels of VVG exposure, participants updated their beliefs to align with the research they read, regardless of whether it suggested VVGs increase or decrease aggression. However, this alignment weakened and disappeared as VVG exposure increased in the “Positive effect” condition.

Notably, at very high levels of VVG exposure (≥17), participants exposed to research indicating that VVGs increase aggression actually updated their beliefs in the opposite direction. These findings suggest that at high exposure levels, not only were the abstracts ineffective, they were even counterproductive. Moreover, the exploratory analyses suggest that the critical feature of the belief update in the “Positive Effect” condition is *whether* participants align with the claim “VVGs increase aggression” or distance themselves from it. Figure 4 reveals a color-gradient pattern: arrows shaded red (indicating high VVG exposure) seem to be concentrated in the lower portion of the plot and predominantly point leftward, whereas blue arrows (low exposure) seem to cluster higher up and more often point rightward. Figure 5 reveals how the cumulative probabilities of aligning and distancing change as a function of VVG exposure. Taken together, the pattern shows that the more habitual a player is, the more the reading pushed them to distance themselves from the claim that VVGs increase aggression. This illustrates the result of the exploratory analysis which suggested that each unit increase in VVG exposure raises the odds of distancing (vs. alignment) by 14 %.

Finally, the model comparisons suggest that the precise endpoint of the updated belief—whether it lands at the “null” belief (i.e. a belief in no effect) or crosses into a “negative” belief (i.e. a belief that VVGs reduce aggression)—does not signal a different phenomenon. What matters is the binary shift away from, or toward, the communicated claim (i.e. the claim that “VVGs increase aggression”). Of note, our pre-registered hypothesis was conservatively formulated, predicting *only* that VVG players would persist in believing that VVGs do not increase aggression, irrespective of the research conclusions. Given our finding, we deemed it essential to carry out a second study to determine whether this effect would replicate.

## Study 2

A potential concern with Study 1 is that participants with higher VVG exposure might use their domain knowledge to critically assess the research. To address this and rule out domain knowledge as the primary influence rather than motivated reasoning, Study 2 aimed to replicate Study 1 with a key change: using standardized fictional article abstracts that varied only in their reported findings and conclusions.

Additionally, to ensure that the findings were not skewed by the potentially technical nature of the abstracts in Study 1, Study 2 included a standardized press article that summarized the findings in lay terms.

### Method

The sampling and recruitment process and rationale were identical to study 1. Study 2 was conducted online, from October 15th, 2023 to October 16th, 2023.

The *N* = 788 participants were from 29 different countries. The mean age of the sample was *M* = 35.2 (*SD* = 12.49), *n* = 339 were females, *n* = 448 were males, and *n* = 1 reported being non-binary.

**Figure 6.**
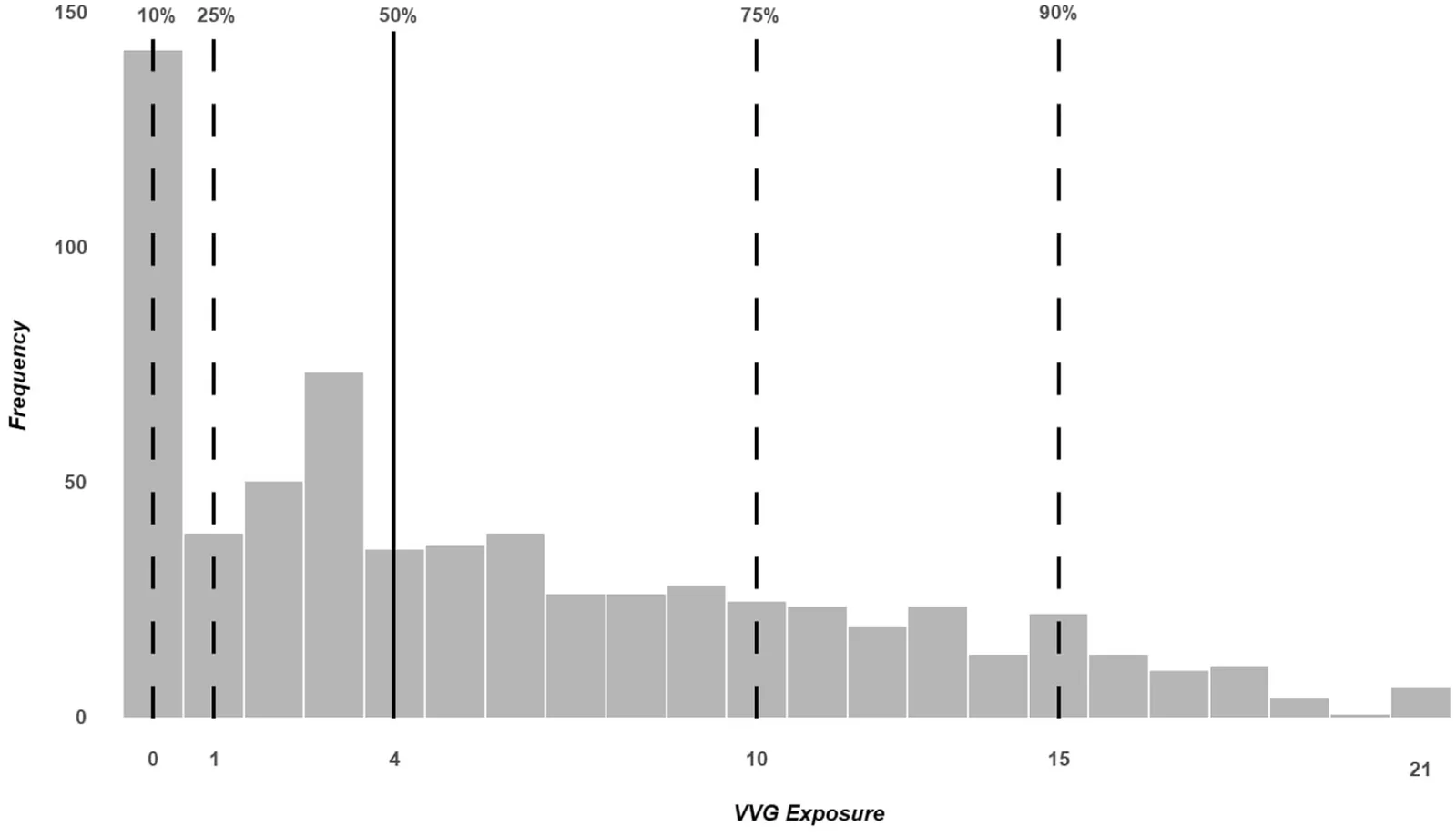
Histogram of the distribution of VVG exposure scores (in Study 2). *Note*. The solid vertical line represents the median value and the four dashed lines represent the 10th, 25th, 75th, and 90th percentiles.

To assess VVG exposure, we used the same measure as we did in Study 1. The average level of VVG Exposure was *M* = 5.93 (*SD* = 5.49; see Figure 6 for the full distribution).

The presentation of the task was similar to Study 1, but included an additional element. Participants were informed they would review three types of scientific communications rather than two.

Participants were randomly placed into either the “Positive effect” condition (*n* = 395), where a press article, a meta-analysis, and a primary study all suggested that VVGs increase aggression, or the “Null effect” condition (*n* = 393), where the three documents indicated no effect of VVGs on aggression.

Three fictional documents were created, with fictional author names. These documents, along with the titles, study description and writing style, were designed to be as realistic as possible:

- *Meta-analysis*: All documents presented were fictional, with invented author names and findings. Participants in both conditions were presented a meta-analysis titled “*The Effects of Violent Video Games on Aggressive Outcomes: A Meta-Analysis*,” authored by fictional authors *E. Wong, D. Gent, P. Markey, K. Durbin*, and *J. Baker*. This meta-analysis purportedly reviewed 280 effect sizes from 138 studies comparing VVG players with non-players. Several details were included to increase the credibility of the text. The meta-analysis was described as using a random effects meta-regression with robust variance estimates and three publication bias methods to address selective reporting, noting significant heterogeneity with an I² of 50% and a *p*-value of .003. The “Null effect” condition’s abstract concluded no significant VVG impact on aggressive thoughts, feelings, or behaviors, thereby refuting the hypothesis that VVGs increase aggression. Conversely, the “Positive effect” condition’s abstract found a moderate significant effect of VVGs on these outcomes, supporting the hypothesis that VVGs increase aggression.
- *Experimental study*: A fictional experimental study titled “*From Pixels to Reality? An Experimental Laboratory Study of the Effects of Video Game Violence on Aggressive Behavior*” by *E. Wong* and *J. Baker* (the meta-analysis’s lead and last authors) was also presented in both conditions. This study involved 96 psychology undergraduates, randomly assigned to play either a neutral game or a VVG, with aggression measured via a modified hot sauce paradigm. In the “Null effect” condition, results showed no increase in aggression among VVG players, challenging the General Aggression Model (GAM). In the “Positive effect” condition, findings indicated increased aggression among VVG players, supporting the GAM.
- *Press article*: A fictional press article by a fake journalist *Steven Donner* summarized the findings from the two studies, employing clickbait-style accessible language. It portrayed interviews with fictional researchers *Karen Durbin* and *Emily Wong* to explain the results and implications straightforwardly. In the “Null effect” condition, the article emphasized the null findings, starting with the headline “*Violent video games: do they make you more aggressive? No!*” In the “Positive effect” condition, it affirmed the link between VVGs and aggression, highlighted by the headline “*Violent video games: do they make you more aggressive? Yes!*” and several statements within the article, including the direct quotes from the fictional authors.

Importantly, the methodological aspects of the meta-analysis and experimental study remained identical across both conditions, ensuring that any differences in participant reactions were due to the outcome presentations rather than methodological discrepancies.

To assess the “believed relationship between VVGs and aggression” and belief update, we used the same measure as we did in Study 1. The average level of belief update was *M* = −0.02 (*SD* = 0.14).

### Results

#### Pre-Manipulation and Post-Manipulation Beliefs

**Figure 7.**
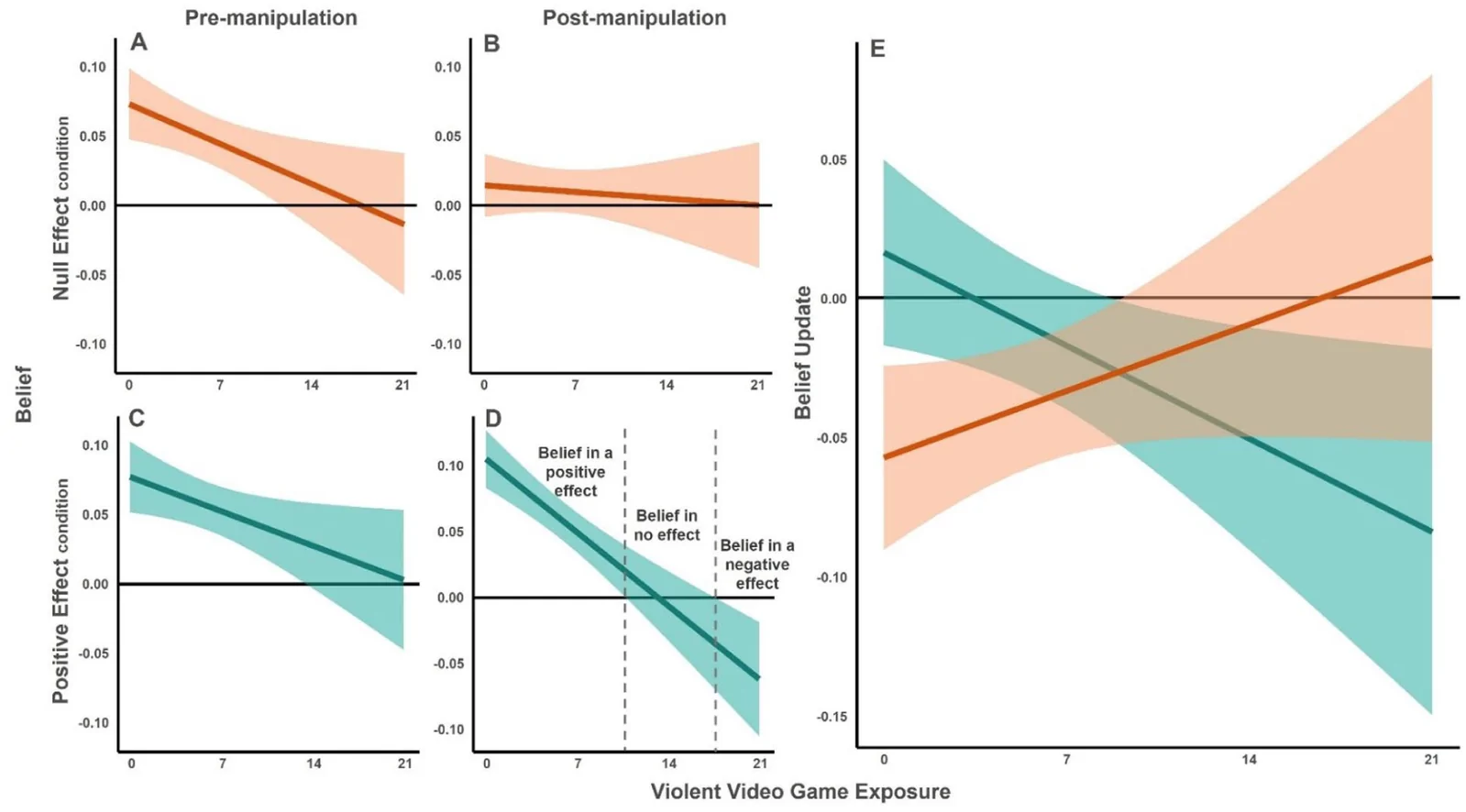
The relationship between VVG exposure, belief, and belief update in the two conditions (in Study 2). *Note.* The *x*-axis in the five panels corresponds to participants’ VVG exposure. The *y*-axis on panels A, B, C, and D corresponds to the belief about the relationship between VVG exposure and aggressivity. The *y*-axis in panel E corresponds to the belief update (i.e. the post-manipulation beliefs minus the pre-manipulation beliefs). The thick horizontal line in the five panels corresponds to the zero threshold. Panel A shows participants’ pre-manipulation belief, as a function of VVG exposure, in the “Null effect” condition (Std. β = −.21). Panel B shows participants’ post-manipulation belief, as a function of VVG exposure, in the “Null effect” condition (Std. β = −.04). Panel C shows participants’ pre-manipulation belief, as a function of VVG exposure, in the “Positive effect” condition (Std. β = −.18). Panel D shows participants’ post-manipulation belief, as a function of VVG exposure, in the “Null effect” condition (Std. β = −.45). Panel D shows the belief update, as a function of VVG exposure, in “Null effect” condition (Std. β = .14) and the “Positive effect” condition (Std. β = −.19). The bands in all five panels correspond to the Bonferroni-adjusted-95% CIs.

The average level of pre-manipulation belief was *M* = 0.05 (*SD* = 0.11), with practically identical levels in the “Positive effect” condition (*M* = 0.06, *SD* = 0.12), and the “Null effect” condition (*M* = 0.05, *SD* = 0.09), and statistically equivalent levels between the two conditions, as indicated by the Two-Sided Two-Sample Welch *t*-test, *t*(706.47) = −0.99, *p* = .325, Cohen’s *d* = −0.07, 95% CI [−0.22, 0.07], and a significant Two-One-Sided-Test for equivalence bounds from Cohen’s *d* = −0.05 to Cohen’s *d* = 0. 05, *t*(706.47) = 6.04, *p* < .001. However, unlike Study 1, the pre-manipulation belief was significantly and negatively related to VVG exposure, *t*(786) = −5.59, *p* < .001, Std. β = −.20, 95% CI [−0.26, −0.13] (see Figure 7).

The average level of post-manipulation belief was *M* = 0.03 (*SD* = 0.10), but with significantly different levels in the “Positive effect” condition (*M* = 0.04, *SD* = 0.12), and the “Null effect” condition (*M* = 0.01, *SD* = 0.08), as indicated by a Two-Sided Two-Sample Welch *t*-test, *t*(680.22) = −4.58, *p* < .001, Cohen’s *d* = −0.35, 95% CI [−0.50, −0.20]. The post-manipulation belief was also significantly related to VVG exposure, *t*(786) = −7.02, *p* < .001, Std. β = −.24, 95% CI [−0.31, −0.18]. Moreover, the post-manipulation belief was predicted by the interaction of VVG exposure and the two conditions, *t*(784) = −6.24, *p* < .001, Std. β = −.42, 95% CI [−0.55, −0.29] (see Figure 7). The simple slope for the relationship between VVG exposure and post-manipulation belief in the “Positive effect” condition was *t*(784) = −9.59, Bonferroni-adjusted- *p* < .001, Std. β = −.45, 95% CI [−0.55, −0.34]. The simple slope for the relationship between VVG exposure and post-manipulation belief in the “Null effect” condition was *t*(784) = −.79, Bonferroni-adjusted- *p* = .863, Std. β = −.037, 95% CI [−0.14, 0.07]. In the “Null effect” condition, the post-manipulation belief never differed from zero (all |*t*| < 3, adj. ps > .20). In the “Positive effect” condition, the post-manipulation belief was above zero for VVG exposure levels of 0 to 9, indistinguishable from zero from VVG exposure levels of 10 to 14, and below zero from VVG exposure 15 onward.^10^

The pre-manipulation belief and the post-manipulation belief were correlated at Pearson’s *r* = .11, 95% CI [0.04, 0.18], *t*(786) = 3.05, *p* = .002.

#### Pre-Registered Analysis: The Moderation Effect of VVG Exposure and Article Conclusion on Belief Update

We employed a linear regression model with an interaction term between the condition variable and VVG exposure, and with belief update as the outcome variable. In support of our hypothesis, the interaction between the condition variable and VVG exposure was significantly related to belief update, *t*(784) = −4.61, *p* < .001, Std. β = −.32, 95% CI [−0.46, −0.19] (see Figure 7). The simple slope for the relationship between VVG exposure and belief update in the “Positive effect” condition was *t*(784) = −3.79, Bonferroni-adjusted- *p* < .001, Std. β = −.19, 95% CI [−0.30, −0.07]. The simple slope for the relationship between VVG exposure and belief update in the “Null effect” condition was *t*(784) = 2.72, Bonferroni-adjusted- *p* = .013, Std. β = .14, 95% CI [0.02, 0.25]. In the “Null effect” condition, only at low levels of VVG exposure did participants update their beliefs (VVG exposure levels of 0–9), and they did so downward. For participants with higher levels of VVG exposure (10 or above), the belief update was statistically indistinguishable from zero. In the “Positive effect” condition, for participants at low levels of VVG exposure (0–8), belief update was statistically indistinguishable from zero, however, with higher levels of VVG exposure (9 or above), participants updated their belief downward.^11^

#### Exploratory Analyses

To examine which type of conceptualization best describes the shifts in belief in the “Positive Effect” condition, we repeated the three-approach procedure used in Study 1—with Shift-Trajectory (9 paths), Shift-Direction (3 directions), and Shift-Distancing (aligned vs. distanced)—in the “Positive effect” condition (*n* = 395). As in Study 1, the simple binary model provides the best trade-off between parsimony and fit; adding finer outcome granularity only inflates information criteria and error (see Table 3).

**Table 3.** Model Comparison Metrics.

Model	Pattern	AICc	Δ AICc	BIC	RMSE	Residual σ
m1	*Shift-trajectory*	1,419	883	1,481	7.51	1.91
m2	*Shift-direction*	818	282	834	2.49	1.44
m3	*Shift-distancing*	536	0	544	2.05	1.16

**Figure 8.**
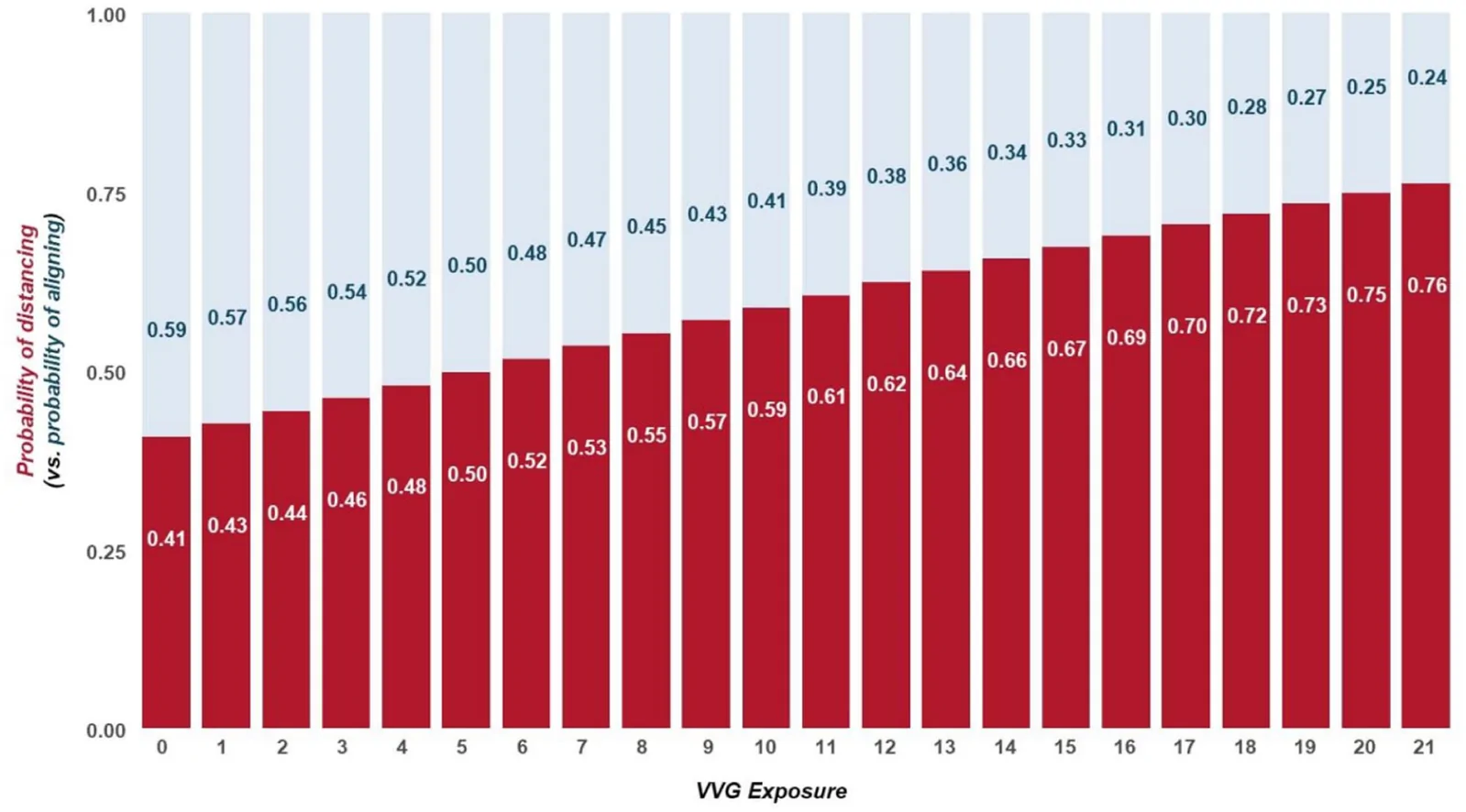
The probability of distancing from the claim (vs. the probability of aligning) as a function of VVG exposure. *Note*. The figure displays the predicted probability of participants distancing from the claim (vs. aligning with it) as a function of VVG exposure, among participants assigned to the “Positive effect” condition (*n* = 395). Predicted probabilities are shown for each integer level of VVG exposure (0–21). Probabilities were derived from a binary logistic-regression model in which VVG exposure was entered as a continuous predictor of distancing from the claim (reference category = aligning). For every exposure level, the fitted logit was first calculated and then converted to a probability with the inverse-logit transformation. The model yielded an odds ratio of 1.08 (*p* < .001), indicating an 8% increase in the odds of distancing for each additional unit of VVG exposure. Numeric labels inside the bars are the rounded predicted probabilities.

The results of the *Shift-Distancing* model indicated that each unit increases in VVG exposure increases the odds of distancing from the “VVGs increase aggression” claim by 8% (*OR* = 1.08, *p* < .001). Hence, at a VVG exposure level of 0, the probability of aligning to claim is 59% versus a 41% probability of distancing, whereas at a VVG exposure level of 21, the probability of aligning is 24% versus a 76% probability of distancing (see Figure 8).

### Discussion

Study 2 replicated all the main findings from Study 1. Specifically, at high levels of VVG exposure, individuals who initially believed that VVGs have no effect on aggression developed a new belief that VVGs actually *decreased* aggression after reading evidence to the contrary. Importantly, the use of standardized fictional article abstracts in Study 2 indicates that these effects cannot be solely attributed to methodological or stylistic variations. As in Study 1, the binary “alignment versus distancing” conceptualization captured the belief update pattern most efficiently: knowing whether participants ended up believing that VVGs have no effects or that they decrease aggression added complexity with no explanatory value.

## General Discussion

We hypothesized that at low levels of VVG exposure, participants in both conditions would update their beliefs in the direction of whichever research they were reading, and that at higher levels of VVG exposure, participants would maintain their belief that VVGs do not increase aggression, regardless of the condition. Our two studies support this moderation hypothesis. Study 1, which used published articles, shows that our observed effect is ecologically valid. Study 2, which used standardized fictional articles, demonstrates that the effect is not due to variations in research methodology, writing style, or quality, but is caused by the research conclusions.

Across both studies, a consistent homogeneous set of scientific claims convinced readers to align their beliefs only when they had little personal stake. Non-gamers and participants who had low levels of VVG exposure shifted their beliefs toward the conclusion of the research that they read, however, habitual players did not. Specifically, at very high levels of VVG exposure, reading research suggesting that VVGs increase aggression led participants to believe the opposite: that VVGs actually *reduce* aggression, a notion known as the “belief in catharsis” (see Gentile, 2013; Kersten & Greitemeyer, 2022). The pattern replicated with real abstracts (Study 1) and fully standardized fictional materials (Study 2). This indicates that message homogeneity alone is not enough to persuade an audience that feels threatened.

Our findings conceptually replicate and extend those of Anglin (2019) and Rosman and Grösser (2024), who found that exposure to a unidirectional set of scientific claims led participants to update their beliefs in line with the presented evidence. Across both of our studies, we observed a similar pattern—but *only* among participants for whom the topic was not personally threatening. For individuals with high exposure to VVGs, belief updating was significantly reduced or even reversed, highlighting the importance of motivational stakes in moderating the persuasive power of coherent scientific messages. Furthermore, compared to the studies by Anglin and Rosman and Grösser, our methodology likely reduced the influence of demand characteristics. Whereas prior work used direct, repetitive self-report items to measure belief change—raising the likelihood of participants guessing the study’s aim—our belief measure was subtler, based on scenario judgments and individually estimated regression slopes that more directly assessed beliefs, reduced repetitiveness, and minimized transparency.

The exploratory analyses in both studies and the model selection results help interpret our findings. In both studies, a binary conceptualization—that is, whether participants shifted their beliefs toward the claim or away from it—outperformed more complex conceptualization that accounted for where the post-manipulation belief was. These results suggest that the fact that participants ended up believing in a negative (i.e. cathartic) effect of VVGs rather than a “null” effect should perhaps not be interpreted as a distinct characteristic of the phenomenon. Specifically in both studies, accounting for “where” the post-manipulation belief was resulted in poorer model fits due to lower parsimony. While it does have qualitative value for its practical implications and societal discourse, the psychological phenomenon we demonstrate here is not necessarily one that results in generating opposite beliefs, but rather one that results in distancing from a claim. Among those who do distance their belief from the claim, there is variability in where their pre-manipulation belief was, and in how much distancing they manifest, which ultimately influences whether some will end up believing in a null effect and others in a cathartic effect—but in both cases, the phenomenon likely remains the same.

While we operationalized this within the context of VVG research and players, this likely represent a broader issue. Various combinations of research topics and audiences can similarly evoke reactance or stigmatization, potentially undermining the intended impact of the research. Even within VVG research, researchers themselves could be a reactant group, as evidenced by the persistent lack of consensus, further highlighting the need to minimize the threatening aspects of scientific research.

A growing body of research on science communication offers concrete strategies to reduce defensiveness among motivated audiences, such as habitual VVG players. Several approaches have been shown to mitigate perceived threats and facilitate more open engagement with contested findings. For example, using autonomy-supportive language and appending a brief reminder of choice can reduce perceived coercion and lower psychological reactance (Richards et al., 2021). Two-sided messages that acknowledge and refute a counterargument can increase perceived credibility and reduce suspicion that information is being selectively presented (Banas & Rains, 2010; O’Keefe, 1999). Similarly, narrative reframing—presenting scientific messages through concrete, human-centered stories—can increase comprehension and engagement without triggering defensiveness (Dahlstrom, 2014). Moreover, preemptive inoculation techniques that warn audiences of common misinformation tactics and provide brief refutations have been shown to protect beliefs from later distortion, even in politically polarized contexts (van der Linden et al., 2017). To reduce dissonance and protect self-concept, interventions such as self-affirmation—where individuals reflect on core personal values—have been shown to increase openness to counter-attitudinal evidence (Petty & Krosnick, 2014; van Prooijen & Sparks, 2014). When scientific information threatens self-worth or competence, value-congruent reframing can reduce resistance—for example, presenting evidence acceptance as a signal of rationality, integrity, or curiosity (Sherman & Cohen, 2006). Social identity threats can be alleviated by using in-group messengers and aligning messages with the audience’s cultural or moral worldview—a strategy known as moral reframing (Feinberg & Willer, 2013, 2015). Additionally, the perceived warmth and trustworthiness of the communicator plays a crucial role: audiences are more receptive to messages from sources they view as empathetic and socially motivated (Fiske & Dupree, 2014; Kraft-Todd et al., 2017; Pornpitakpan, 2004). In highly polarized or identity-relevant domains such as vaccination and climate change, researchers have found that these approaches—particularly source alignment and value-based framing—enhance message credibility and reduce defensive processing (Hornsey & Fielding, 2017; Nisbet & Scheufele, 2009).

### Limitations, Implications, and Future Directions

Our studies have some noteworthy limitations. First, we did not explicitly measure the perception of threats, nor the motivations of participants. This consists in both a limitation and an advantage. On the one hand, not measuring the perceived threat or the motivation does not allow us to provide formal empirical evidence of the causal chain underlying the mechanism we describe. On the other hand, using a proxy (i.e. a subtle measure of VVG exposure which is theorized to be indicative of the threats and the motivation to be defensive) likely helped protect our design from demand effects allowing us to capture more precise and subtle shifts in belief update.

Moreover, we recognize that heavy gamers may be drawing from personal experience, which could make them legitimately skeptical of broad claims that VVGs increase aggression. This may reflect a threat to internal consistency (Festinger, 1957). After years of play, they may hold a large store of personal memories in which nothing obvious suggests that gaming made them more aggressive—or, if any changes did occur, they could not objectively evaluate their own relative aggressiveness against an objective standard. When a few scientific abstracts claim that VVGs raise aggression, players have to choose between (a) their subjective interpretation of their everyday experience and (b) the new research reports. In such a context, personal experience may win because it would feel more vivid, and may be considered like direct evidence, whereas journal articles would be seen as abstract and distant. Long-time players may also see themselves as experts on the topic, further increasing their skepticism toward studies that contradict what they believe they know. In this way, the VVG field is especially challenging: those with the greatest motive to defend gaming also believe they have first-hand knowledge that outweighs scientific data. Our studies cannot rule out this experience-based skepticism. However, our data may reflect both domain knowledge and motivated reasoning. Real experience can inform beliefs, yet the polar-opposite shift we observed suggests that identity or reactance processes also play a role. Future research might measure and experimentally manipulate participants’ sense of domain expertise to parse these effects. Study 2 employed fictional but methodologically standardized abstracts, suggesting that differences in methodology or writing style are *not* the main drivers. However, it remains possible that heavy gamers doubted these results because the manipulations diverged from their lived experiences with VVGs.

Similarly, participants’ resistance to certain claims could be normatively reasonable if they correctly perceive such abstracts as unrepresentative of the broader literature, or if their personal observations diverge from the conclusion presented. Therefore, not all resistance should be attributed to irrational bias. However, their evaluation of the representativeness of these studies could itself be biased by motivated reasoning. Future studies might compare participants’ self-reported knowledge of the research debates—or measure the perceived realism/credibility of the studies—to gauge this more precisely. We also acknowledge that heavy VVG players might differ in demographic variables (e.g. education), or in their overall trust in science. Although random assignment and large sample sizes help mitigate this concern, future work should include explicit measures of scientific trust, educational background, and gaming culture knowledge to disentangle these factors from motivated reasoning. Even if heavy gamers have reasons to doubt certain findings, the strength of the observed effect—shifting to believe that VVGs *reduce* aggression—fits well with the concept of motivated reasoning. Personal experience alone does not necessarily mandate a polar-opposite shift; hence, it is plausible that domain knowledge *and* psychological reactance work together to produce the observed pattern.

Acknowledging these limitations, our findings can nonetheless be meaningfully interpreted in light of the defensive mechanism of psychological distancing, which offers a coherent account of the belief shifts observed among high VVG exposure participants. Distancing involves moving one’s beliefs away from a dissonant, contradicting or threatening claim and serves as a motivated strategy to reduce discomfort while preserving autonomy, coherence, self-worth, or group loyalty. In our studies, participants with high VVG exposure were not merely resistant to the claim that VVGs increase aggression—they shifted their beliefs in the opposite direction, toward the idea that VVGs reduce aggression. Participants reoriented themselves away from the claim. Importantly, this distancing likely results from the perception of psychological threats, which may fall into four categories: threats to freedom (e.g. feeling pressured to give up a valued activity), threats to consistency (e.g. experiencing dissonance between personal experience and research claims), threats to self-concept (e.g. implications of being immoral or violent), and threats to social identity (e.g. stigmatization of the gamer community). Each of these threats can elicit defensive responses, including reactance, biased assimilation, source derogation, or selective exposure, and we do not rule out the contribution of such mechanisms. Nevertheless, our pattern of results—including the binary structure of belief shifts and the predictive role of personal stake—suggests that distancing was the most central and parsimonious response in this context. Future research could benefit from disentangling the precise conditions under which these defensive strategies co-occur or compete, and how different threats uniquely prompt each type of response.

Beyond its explanatory value in the current context of VVG players and VVG research, the defensive mechanism of psychological distancing that we highlight in this paper also offers a unifying perspective for interpreting a range of belief-related phenomena that have historically been described under disparate labels—such as the backfire effect (e.g. Lewandowsky et al., 2012; Nyhan & Reifler, 2010), boomerang effects (e.g. Hart & Nisbet, 2012), biased assimilation (Lord et al., 1979), and confirmation bias (Nickerson, 1998). While these terms emerged from different paradigms and highlight distinct features of belief resistance, they often describe similar patterns: individuals not only resist corrective information but may also double down on their original stance, or even adopt more extreme counter-positions. By framing these outcomes as manifestations of psychological distancing—where individuals cognitively reposition themselves and their group away from threatening claims—we offer a process-level account that helps integrate these phenomena under a common theoretical umbrella. More specifically, if psychological distancing is occurring, it matters not where the final belief lands but rather that individuals are enhancing the distinction between themselves and the prototypical outgroup that they perceive on the dimension made salient by the statement. This conceptual clarity can aid efforts to disentangle genuinely distinct mechanisms from terminological artifacts, addressing potential jingle–jangle fallacies that complicate cumulative theory-building in the field.

More broadly, although our primary focus was on VVG research, our findings speak to broader issues of stigmatization, reactance, and confirmation bias that may underlie public skepticism toward scientific evidence in various areas. This raises an important question: How can researchers in psychology, and in other fields, effectively communicate their work without reinforcing the very misconceptions they hope to correct? Scholars must consider both the content and the framing of their messages if they wish to have a meaningful societal impact. The framing of research is crucial: poorly framed information may not only fail to dispel misconceptions but may inadvertently intensify them. Future studies should thus explore how individuals across diverse contexts and topics update their beliefs when presented with scientific evidence. Without addressing these crucial points, our well-intentioned scientific endeavors risk not only falling on deaf ears but also backfiring, actively fueling the very misconceptions we aim to dispel.

## Notes

1. In the methods and results sections for both studies, we provide information about the variables relevant to the hypothesis and discussion. The full list of variables and the exploratory analyses are all made available in the supplemental materials. The same applies to Study 2.

2. This approach was fully pre-registered. We provide evidence in support of the validity and performance of this VVG exposure measure in the supplemental materials on the OSF repository.

3. More details are provided in the supplemental materials.

4. Full point-wise tests, Confidence Intervals (CIs), and Estimated Marginal Means (EMMs) appear in the supplemental materials.

5. On average, the belief coefficient before reading the abstracts was 0.03. This means that participants, on average, believed that for each one-unit increase in exposure dosage (for example, playing a game that is 1% more violent for one more hour), aggression would increase by 0.03 points. For example, playing a game that is 100% violent for 1 hr (100 units) would be expected to increase aggression by 3 points. Similarly, a 50% violent game played for 3.5 hr (175 units) would correspond to an increase of 5.25 points. Participants who read a research summary suggesting that VVGs have no effect (the “Null effect” condition) had an average coefficient of 0.01. This means they believed that each one-unit increase in exposure dosage would increase aggression by just 0.01 points. For example, playing a 100% violent game for 1 hr would increase aggression by only 1 point, and a 50% violent game for 3.5 hr would increase aggression by 1.75 points. In contrast, participants who read a summary suggesting a positive effect had an average coefficient of 0.05, meaning they believed that each one-unit increase in exposure dosage would increase aggression by 0.05 points. For them, a 100-unit exposure (100% violent for 1 hr) would increase aggression by 5 points, and a 175-unit exposure (50% violent for 3.5 hr) would increase aggression by 8.75 points.

6. Three other analytic approaches exist for analyzing pre-post data: residual change scores, ANCOVA adjusting for baseline, and mixed-effects modeling. However, we opted for simple change scores (post minus pre), which offer several advantages. With random assignment, baseline differences are random noise, making this approach unbiased. Change scores directly measure how much participants changed on the original scale, simplifying interpretation and communication. Unlike ANCOVA, it does not assume equal pre-post relationships across groups. Further, unless pre-post correlations are very high, the change-score method provides comparable or greater statistical power.

7. Complete point‑wise tests, CIs, and EMMs are provided in the Supplementary Materials.

8. Participants’ who already had a belief > .05 (i.e., positive belief) and didn’t have a decrease and participants who had a belief < .05 (null belief or negative belief) and had an increase were considered congruent: they aligned with the claim that VVGs increase aggression.

9. Note that the -0.05 and 0.05 thresholds were selected arbitrarily as there are no participants with an exact 0 change.

10. Complete point wise tests, CIs, and EMMs are reported in the Supplementary Materials.

11. Complete point‑wise tests, CIs, and EMMs are reported in the Supplementary Materials.
